# Molecular Analysis of Membrane Targeting by the C2 Domain of the E3 Ubiquitin Ligase Smurf1

**DOI:** 10.3390/biom10020229

**Published:** 2020-02-04

**Authors:** Jordan L. Scott, Cary T. Frick, Kristen A. Johnson, Haining Liu, Sylvia S. Yong, Allyson G. Varney, Olaf Wiest, Robert V. Stahelin

**Affiliations:** 1 Department of Chemistry and Biochemistry, University of Notre Dame, Notre Dame, IN 46556, USA; jordanscott28@gmail.com (J.L.S.); cfrick12@gmail.com (C.T.F.); Kristen.Johnson@UTSouthwestern.edu (K.A.J.); hainingliu14@gmail.com (H.L.); syong@alumni.nd.edu (S.S.Y.); allykat1850@gmail.com (A.G.V.); owiest@nd.edu (O.W.); 2 Department of Medicinal Chemistry and Molecular Pharmacology and the Purdue Center for Cancer Research, Purdue University, West Lafayette, IN 47907, USA

**Keywords:** C2 domain, E3 ubiquitin ligase, lipid binding, phosphoinositide, plasma membrane, Smurf1, ubiquitin

## Abstract

SMAD ubiquitination regulatory factor 1 (Smurf1) is a Nedd4 family E3 ubiquitin ligase that regulates cell motility, polarity and TGFβ signaling. Smurf1 contains an N-terminal protein kinase C conserved 2 (C2) domain that targets cell membranes and is required for interactions with membrane-localized substrates such as RhoA. Here, we investigated the lipid-binding mechanism of Smurf1 C2, revealing a general affinity for anionic membranes in addition to a selective affinity for phosphoinositides (PIPs). We found that Smurf1 C2 localizes not only to the plasma membrane but also to negatively charged intracellular sites, acting as an anionic charge sensor and selective PIP-binding domain. Site-directed mutagenesis combined with docking/molecular dynamics simulations revealed that the Smurf1 C2 domain loop region primarily interacts with PIPs and cell membranes, as opposed to the β-surface cationic patch employed by other C2 domains. By depleting PIPs from the inner leaflet of the plasma membrane, we found that PIP binding is necessary for plasma membrane localization. Finally, we used a Smurf1 cellular ubiquitination assay to show that the amount of ubiquitin at the plasma membrane interface depends on the lipid-binding properties of Smurf1. This study shows the mechanism by which Smurf1 C2 targets membrane-based substrates and reveals a novel interaction for non-calcium-dependent C2 domains and membrane lipids.

## 1. Introduction

Ubiquitin ligases regulate processes such as endocytosis, signal transduction, protein degradation, and viral egress [1,2,3,4,5]. The Nedd4 family of E3 ubiquitin ligases has nine members in humans, each featuring an N-terminal protein kinase C conserved 2 (C2) domain, two to four WW domains for substrate protein recognition, and a C- terminal HECT domain for ubiquitin transfer [6]. SMAD ubiquitination regulatory factor 1 (Smurf1) is a Nedd4 family E3 ubiquitin ligase initially identified as an inhibitor of transforming growth factor beta (TGF-β) signaling [7,8,9]. Smurf1 is now known to regulate several other cellular targets, including RhoA, Runx2 and TRAF4 [2,10,11,12]. Furthermore, it helps to maintain cellular homeostasis by regulating processes such as cell polarity, cell transformation and growth, the clearance of damaged mitochondria, and neuronal axon growth [2,3,13,14,15,16].

C2 domains are lipid-binding domains that enable the specific targeting of cell membranes [17], thus allowing proteins to interact with membrane-localized substrates, such as RhoA at the plasma membrane in the case of Smurf1 [10]. These domains are regulated spatially by the intracellular localization of target lipids and can also be regulated temporally by the synthesis of cognate lipids, phosphorylation, or ion influx [18,19]. They contain a canonical eight-stranded antiparallel β-sandwich fold and a loop region composed of three extended loops between β strands (in Smurf1 they are the β1-2, β3-4 and β5-6 loops). Although some C2 domains bind reversibly at cell membranes in response to increases in intracellular calcium, Smurf1 C2 lacks the consensus Ca^2+^-binding residues (Asp, Asn, and/or Glu) required for this mode of regulation. The loops are adjacent to the shallow β-groove region of the protein, which comprises the upper portion of the β2 and β3 strands and regulates binding to phosphoinositides (PIPs) in several known C2 domains, including those of PKCα, Kibra, and synaptotagmins [20,21,22,23].

The Lys28 and Lys85 residues in the loop region of the Smurf1 C2 domain are required for membrane localization and the ability of Smurf1 to target RhoA and hPEM-2 for degradation [10]. The lack of negatively charged residues normally required for the coordination of calcium in the loop region of C2 domains means that the corresponding region of the Smurf1 C2 domain is comparatively much more basic [20]. As such, it is likely that calcium-independent interactions with the plasma membrane are driven mainly by electrostatic bonds between the positively charged protein and the negatively charged membrane, in addition to selective lipid head group recognition and protein–protein interactions. 

Although Smurf1 can target plasma membranes, the lipids bound by the C2 domain and the amino acids involved in the interaction are unknown. To unravel the molecular basis of calcium-independent membrane targeting by the Smurf1 C2 domain, we combined biophysical and cellular assays to determine its lipid binding selectivity. We used centrifugation assays, surface plasmon resonance (SPR) spectroscopy, and confocal microscopy to investigate the binding of the Smurf1 C2 domain in vitro to lipid vesicles and to mammalian cells with PIP-depleted plasma membranes. We also created lipid-binding mutants and analyzed their behavior in vitro and in cells by co-localization with PIP-binding proteins and fluorescent markers of anionic lipids, thus narrowing down the region required for specific interactions. Our results demonstrate that the Smurf1 C2 domain has unique lipid binding properties, acting as an anionic charge sensor with some PIP selectivity for PI(4,5)P_2_ and PI(3,4,5)P_3_, thus enabling Smurf1 to target the plasma membrane and other intracellular sites harboring anionic lipids. 

## 2. Materials and Methods 

### 2.1. Materials

The lipids 1-palmitoyl-2-oleoyl-*sn*-glycero-3-phosphocholine (POPC), 1-palmitoyl-2-oleoyl-*sn*- glycero-3-phosphoethanolamine (POPE), 1-palmitoyl-2-oleoyl-*sn*-glycero-3-phospho-L-serine (POPS), 1-palmitoyl-2-oleoyl-*sn*-glycero-3-phosphoinositol (POPI), 1,2-dioleoyl-*sn*-glycero-3- phospho-(1′-myo-inositol-4′,5′-bisphosphate, 18:1 PI(4,5)P_2_) (PI(4,5)P_2_), 1,2-dioleoyl-*sn*-glycero- 3-phospho-(1′-myo-inositol-3′,4′,5′-trisphosphate, 18:1 PI(3,4,5)P_3_) (PI(3,4,5)P_3_), 1,2-dioleoyl-*sn*- glycero-3-phospho-(1′-myo-inositol-3′,4′-bisphosphate, 18:1 PI(3,4)P_2_) (PI(3,4)P_2_), 1,2-dioleoyl-*sn*- glycero-3-phospho-(1′-myo-inositol-3′-monophosphate, 18:1 PI(3)P) (PI(3)P), 1,2-dioleoyl-*sn*- glycero-3-phospho-(1′-myo-inositol-4′-monophosphate, 18:1 PI(4)P) (PI(4)P), and 1,2-dioleoyl-*sn*- glycero-5-phospho-(1′-myo-inositol-3′-monophosphate, 18:1 PI(5)P) (PI(5)P) were purchased from Avanti Polar Lipids (Alabaster, AL, USA) and used without further purification. The lipids were dissolved in solvents according to the manufacturer’s recommendation and stored as recommended unless specified. Cholesterol from ovine wool was also purchased from Avanti Polar Lipids and used without further purification. Avanti Lipid Snoopers were a kind gift from Walt Shaw (Avanti Polar Lipids).

### 2.2. Smurf1 Full-Length Site-Directed Mutagenesis

Mutations were introduced into the mCherry–Smurf1 full-length construct using the QuikChange II XL site-directed mutagenesis kit (Agilent Technologies, Santa Clara, CA, USA). A549 cells were transfected in serum-free 50/50 Dulbecco’s modified Eagle’s medium/Roswell Park Memorial Institute 1640 (DMEM:RPMI) medium with Lipofectamine LTX (Thermo Fisher Scientific, Waltham, MA, USA) according to the manufacturer’s instructions, using 0.2 μg plasmid DNA, 0.8 μL Lipofectamine LTX and 0.2 μL PLUS reagent. Cells were imaged 24 h post-transfection using a Zeiss LSM710 confocal microscope (excitation wavelength 561 nm, emission wavelength 578–797 nm). Images were acquired in scans of 30–60 s with two-line averaging. The percentage plasma membrane localization (%PM) was determined using a MATLAB script based on the following equation: %PM = average mCherry plasma membrane fluorescence/(average mCherry plasma membrane fluorescence + average mCherry cytoplasmic fluorescence) × 100%.

### 2.3. Protein Purification

The Smurf1 C2 domain with a His_6_-tag was expressed in *Escherichia coli* BL21-DE3 cells and purified using a standard His_6_-tag purification protocol according to the manufacturer’s instructions (Qiagen, Hilden, Germany). Protein expression was induced with 0.15 μM IPTG at 25 °C overnight when the cells reached an optical density at 600 nm (OD_600_) of 0.6–0.8. The cells were then lysed with 0.5 mg/mL lysozyme, vortexed and briefly sonicated in 50 mM NaH_2_PO_4_ (pH 8.0) containing 300 mM NaCl and 1 mM PMSF. The lysate was passed through 0.8 μm and 0.45 μm filters and incubated with Ni-NTA resin (Qiagen) for 1 h at 4 °C on a rocking platform. Beads were then loaded onto a column and washed with 10 column volumes of 10, 20, and 25 mM imidazole in lysis buffer. The protein was eluted using 50 mM NaH_2_PO_4_ (pH 8.0) containing 300 mM imidazole and 300 mM NaCl. The protein was then concentrated and dialyzed using an Amicon Ultra-4 10,000 NMWL regenerated cellulose membrane centrifugal filter (MilliporeSigma, Burlington, MA, USA), quantified using the Pierce BCA assay kit (Thermo Fisher Scientific), and stored in 20 mM HEPES/300 mM NaCl with 1 mM DTT (pH 7.4) at 4 °C for several weeks or at −20 °C with 25% glycerol for several months.

### 2.4. Liposome Binding Assays

Lipid mixtures for pelleting assays were dried under nitrogen and resuspended in 10 mM HEPES, 160 mM KCl (pH 7.4) at a concentration of 1 mg/mL. The solutions were incubated on ice for 2 h and vortexed into multilamellar vesicles (MLVs) until the lipids had detached from the sides of the vials and the solutions were cloudy. The lipids were then incubated with 100 μg/mL protein in 100 μL reactions at room temperature for 20 min followed by centrifugation at 50,000× *g* for 20 min at 4 °C. Supernatants were carefully removed and the pellets were re-suspended in an equal volume (100 μL) of SDS loading buffer. The samples were then fractionated by 12% SDS-PAGE and the bands were quantified by densitometry using ImageJ software (NIH/University of Wisconsin). It should be noted than in these centrifugation assays, the vesicles formed are MLVs as noted above and slightly different than the type of vesicles described in the SPR assays. 

### 2.5. Snooper Lipid Detection

**Figure 1:**
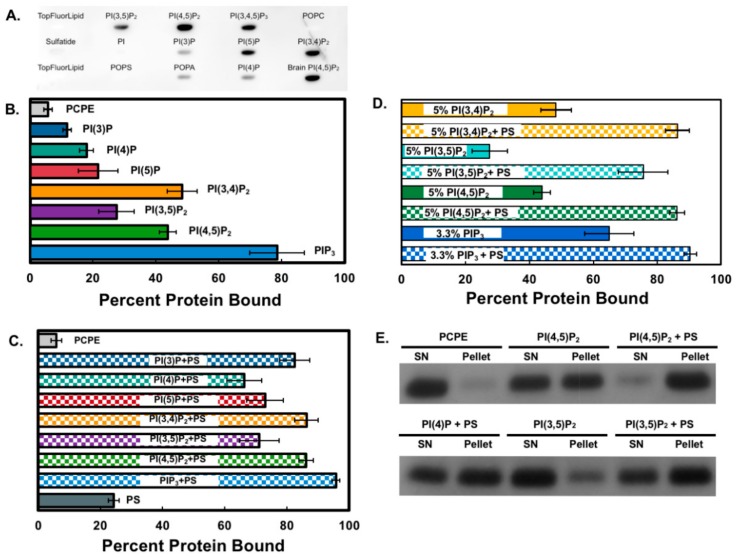
The SMAD ubiquitination regulatory factor 1 (Smurf1) C2 domain binds to phosphoinositides in vitro. (**A**) A protein–lipid overlay assay was performed with a Lipid Snooper® to gauge anionic lipid binding. (**B**) The Smurf1 C2 domain was incubated with multilamellar vesicles (MLVs) at room temperature for 20 min and then centrifuged at 50,000× *g* to separate the supernatant and pellet. Following SDS-PAGE, the percent binding was calculated with respect to control vesicles (80:20, POPC:POPE). MLVs contained 5% of the respective PIPs (75:20:5 PC:PE:PIP). The Smurf1 C2 associated with vesicles containing several PIP species (PI(3,4)P_2_, PI(4,5)P_2_, and PIP_3_). (**C**) Smurf1 C2 binding to PIPs is enhanced in the presence of PS (60:15:20:5, POPC:POPE:POPS:PIP). Checkered boxes indicate the presence of 20 mol% PS. (**D**) Smurf1 C2 binding to vesicles containing PIPs of equimolar charge in the presence and absence of PS. All MLV data are means ± SEM (*n* ≥ 3). (**E**) Example of SDS-PAGE results depicting supernatant (non-lipid-binding) versus pellet MLV (lipid-binding) Smurf1 C2.

Avanti Lipid Snoopers^®^ containing PIPs and several other lipids (Figure 1) were blocked for 1 h with 5% fatty acid-free BSA before incubating with 5 μg/mL protein overnight at 4 °C. After washing, the lipid-containing blots were incubated with a His_4_-specific primary antibody (Qiagen, diluted 1:500) and horseradish peroxidase (HRP)-conjugated secondary antibody (diluted 1:2500). The binding of the Smurf1 C2 domain to different lipids on the Snoopers was then revealed by adding the HRP substrate and imaged by chemiluminescence.

### 2.6. SPR Spectroscopy

Large unilamellar vesicles (LUVs) were prepared for SPR spectroscopy by extrusion through 100-nm filters using an Avanti lipid extruder. LUVs formed by this method contain a bilayer mimetic of cellular membranes and have a more uniform size than the MLVs used above due to extrusion through a 100 nm membrane. LUVs are also captured intact as vesicles on the L1 sensor chip surface, providing a means to monitor binding of proteins to membranes with similar diameter and curvature. All SPR measurements were performed at 25 °C on a Biacore X instrument (GE Healthcare, Chicago, IL, USA). Briefly, 75 μL of protein solution was injected onto a lipid-coated L1 chip at a flow rate of 5 μL/min. Dissociation was measured for 150 s. Protein was removed from the lipid-coated chip by applying brief 40 μL washes of 50 mM NaOH at a flow rate of 100 μL/min. Sensograms were corrected for refractive index changes and non-specific binding by subtraction of the POPC:POPE (80:20) control surface channel from the second channel (lipid mixture of interest). Maximum responses (*R*_eq_, saturation values) were determined and plotted against protein concentrations in KaleidaGraph. The apparent affinity (*K*_d_) of the interaction was determined using a nonlinear least-squares analysis of the binding isotherm: *R*_eq_ = *R*_max_/(1 + *K*_d_/[P]), where [P] is the concentration of protein. Kinetic characteristics were determined by separate analysis of the association and dissociation rates using BiaEvaluation software (GE Healthcare).

### 2.7. Cloning and Plasmids

The Smurf1 C2 pET28-MHL plasmid for protein purification was a kind gift from Dr. Sirano Dhe-Paganon (Structural Genomics Consortium, Toronto, ON, Canada). Enhanced green fluorescent protein (EGFP)-Smurf1 C2 and mCherry–Smurf1 C2 were constructed by transferring the Smurf1 C2 gene from pET28-MHL to the Xho1 and EcoR1 sites of pmEGFP-C1 (plasmid #36412 from Addgene, Watertown, MA, USA) and pmCherry-C1 (Takar Bio, Kusatsu, Japan). The QuikChange II XL site-directed mutagenesis kit was used to generate full-length Smurf1 or isolated C2 domain point mutations in both vectors. Primers were designed according to the manufacturer’s instructions. All clones selected for plasmid DNA preparation were confirmed by sequencing. Pseudojanin, PJ-Sac, PJ-INPP5E and PJ-DEAD were kind gifts from Dr. Gerald Hammond and Dr. Tamas Balla (University of Pittsburgh and NIH. respectively). Akt PH-mRFP and PLCδ PH-mRFP were kind gifts from Dr. Tamas Balla. The lactadherin C2 domain plasmid was provided by Dr. Sergio Grinstein (University of Toronto, Toronto, ON, Canada).

### 2.8. Cell Culture and Transfection

HeLa, HEK293 and COS-7 cells were cultured in DMEM supplemented with 10% FBS and 1% penicillin/streptomycin. They were passaged as needed by washing with Versene followed by dissociation with trypsin (Thermo Fisher Scientific). A549 cells were maintained in 50/50 DMEM:RPMI, and CHOK-1 cells were maintained in 50/50 DMEM:Ham’s F12 (Thermo Fisher Scientific), in both cases supplemented as above. Cells were plated at a density of ~70% in Lab-Tek II Chambered Coverglass slides (Thermo Fisher Scientific) for transfection 24 h later. HEK293, HeLa, A549 and CHOK-1 cells were transfected with Lipofectamine LTX in Opti-MEM (Thermo Fisher Scientific) using 0.2–1.0 μg DNA per well depending on the plasmid, with 1.25 μL Lipofectamine LTX and 1.25 μL PLUS reagent. COS-7 cells were transfected with 3 μL Lipofectamine 2000 and 1 μg total DNA per well in Opti-MEM. Transfections were carried out in a total volume of 450 μL/well. Cells were imaged 12–24 h post-transfection.

### 2.9. Microscopy and Live Cell Imaging

Live cell images were captured on a Zeiss LSM710 confocal microscope with a 63x, 1.4 numerical aperture oil-immersion objective. Smurf1 C2 wild-type and co-localization images were collected in serum-free Opti-MEM unless otherwise stated, in scans of 30–60 s with two-line averaging. Pharmacological treatments were applied to cells by adding 10-fold working solutions of reagent in 50 μL medium. Phenylarsene oxide, wortmannin, ionomycin, and rapamycin stocks were all dissolved in DMSO. We applied 10 μM phenylarsene oxide for 30 min at 37 °C, 100 μM wortmannin for 15 min at 25 °C, and 10 μM ionomycin in freshly-made buffer (20 mM HEPES (pH 7.4), containing 140 mM NaCl, 5 mM KCl, 1 mM MgCl_2_, 100 μM EGTA and 0.7 mM CaCl_2_) for 10 min at 25 °C. Immediate effects (0.5–2 min post-application) were imaged directly. Rapamycin experiments were performed by establishing a baseline image and then applying 100 nM rapamycin in Opti-MEM. Images acquired during these time-lapse experiments were collected as 5 s scans over a period of 5 min. Two scans were acquired, first a CFP (plasma membrane anchor) and mCherry (phosphatase) dual-image scan, and subsequently an EGFP (protein of interest) scan.

### 2.10. Quantitative Image Analysis

Rapamycin images were analyzed in ImageJ. Stacks of images were loaded into ImageJ for each time-series experiment on an individual cell. Images were thresholded and then converted into binary data. The two outermost pixels were used to generate a cellular mask of the plasma membrane. This was achieved by twice eroding the initial binary cellular image and then duplicating the image and dilating it twice. The eroded image was then subtracted from the binary image to generate an annulus around the cell periphery corresponding to plasma membrane fluorescence. The mean pixel intensity in the plasma membrane was calculated for the baseline image before adding rapamycin and for the image at 5 min post-treatment (*I_PM_*). Cytosolic fluorescence for these time points was also calculated by measuring the mean pixel intensity in two regions of each cell (*I_Cyt_*). The PM index (*I_PM_*/*I_Cyt_*) was used to calculate the dissociation index as the ratio of the PM indices at times 0 and 5 min, as previously described [24].

### 2.11. Live Cell Ubiquitination Assay

HEK293 cells in serum-free DMEM were transfected using Lipofectamine LTX according to the manufacturer’s instructions. Mono-transfected wells received 0.3 μg plasmid DNA, 0.5 μL Lipofectamine LTX and 0.3 μL PLUS reagent, whereas co-transfected wells received 0.3 μg of each plasmid with 1 μL Lipofectamine LTX and 0.6 μL PLUS reagent. Cells were imaged 24 h later using a Zeiss LSM710 confocal microscope with excitation wavelengths of 514 nm (EYFP) and 561 nm (mCherry/mRFP) and emission wavelengths of 519–575 nm (EYFP), 604–797 nm (mCherry) or 612–797 nm (mRFP). Images from all channels were acquired sequentially with 30 s scans per channel and two-line averaging. Acquired images were quantified using ImageJ software. The plasma membrane was selected by applying the threshold command to delineate the perimeter of each cell. The binary image was then eroded two to four times depending on the zoom (~400 nm) to define the inner plasma membrane. These two boundaries were selected to represent the plasma membrane of the cell. Likewise, the area enclosed by the inner plasma membrane was selected to define the cytoplasm. The %PM localization was calculated as described above.

### 2.12. Docking and Molecular Dynamics Simulations

The crystal structure of the human Smurf1 C2 domain (PDB code: 3PYC, 1.96 Å resolution) was prepared using the protein preparation wizard in the Schrödinger Suite [25] with a set pH of 7.0. The initial poses of inositol(1,3,4,5)P_4_, inositol(1,4,5)P_3_ and inositol(1,4)P_2_ in Smurf1 were obtained by docking the ligands into the protein using Glide [26]. The environment of the sulfate ion in the Smurf1 crystal structure, which reasonably models phosphate, was used to define the binding pocket. These initial models were simulated by molecular dynamics using Amber14 [27] to allow relaxation of the rigid structure from the initial docking. Although this provides a more realistic description of the interactions between the human Smurf1 C2 domain and the ligand, the time scale of the simulation is too short to allow for the interconversion of different starting orientations of the ligand. The ff03.r1 all-atom force field was used for the protein, whereas the generalized Amber force field (GAFF) [28] was used for the ligands, with restrained electrostatic potential (RESP) charges based on the B3LYP/6-31G* optimized geometries. The protein–ligand complexes were solvated using the TIP3P water model in a periodic box that extended at least 10 Å from the protein surface. The whole system was neutralized by adding chloride ions. After minimization, the system was gradually heated to 300 K and equilibrated at 1.0 atm and 300 K for 400 ps. A 200 ns production run was performed with a time step of 0.002 ps at 1.0 atm and 300 K under NPT conditions. Langevin dynamics with a collision frequency of 1.0 ps^−1^ were used to control the temperature, and isotropic position scaling was used to maintain the pressure [29]. The non-bonded cutoff distance was set to 10 Å. The SHAKE algorithm [30] was used to constrain all bonds that contain hydrogen. The molecular dynamics simulations were performed under periodic boundary conditions, and the particle mesh Ewald (PME) method [31] was used to treat long-range electrostatic interactions.

## 3. Results

### 3.1. The Smurf1 C2 Domain Binds PIPs and Phosphatidylserine In Vitro

To determine how Smurf1 targets membranes and can thus ubiquitinate membrane-localized substrates, we investigated the ability of its C2 domain to bind specific lipids. Preliminary analysis using lipid blots indicated the affinity of Smurf1 C2 for several PIP species (Figure 1), specifically the bisphosphate and trisphosphate forms. In order to assess the binding specificity using lipid vesicles, MLV centrifugation assays were carried out in the presence of all PIP species, which were incorporated into the vesicles at 5 mol%. On average, Smurf1 C2 showed 20% and 40% stronger binding to PIP_3_ compared to the monophosphate and bisphosphate forms, respectively (Figure 1A). Interestingly, the addition of 20 mol% phosphatidylserine (PS) to the vesicles enhanced the binding of Smurf1 C2 to all PIP species (Figure 1B). 

We assessed the specificity of binding to bisphosphate PIPs and PIP_3_ by generating vesicles of equivalent PIP charge, but with the bisphosphate PIPs at 5 mol% and PIP_3_ at 3.3 mol% (Figure 1C). Under these conditions, Smurf1 C2 again showed the strongest binding to PIP_3_ in the absence of PS, but the addition of PS abolished the differences between the bisphosphate PIPs and PIP_3_. Notably, Smurf1 C2 showed only weak binding to PS in the absence of PIPs compared to the PIP + PS mixture (only 25% C2 domain binding in vesicles containing 20 mol% PS). These results demonstrate that the Smurf1 C2 domain can selectively associate with the phosphorylated PIP head group, indicating that the domain may recognize several PIP species when combined with the anionic lipid PS, and that the membranes with these characteristics (containing PIPs and PS) may provide the lipid signals for Smurf1 C2 binding to biological membranes. 

**Figure 2:**
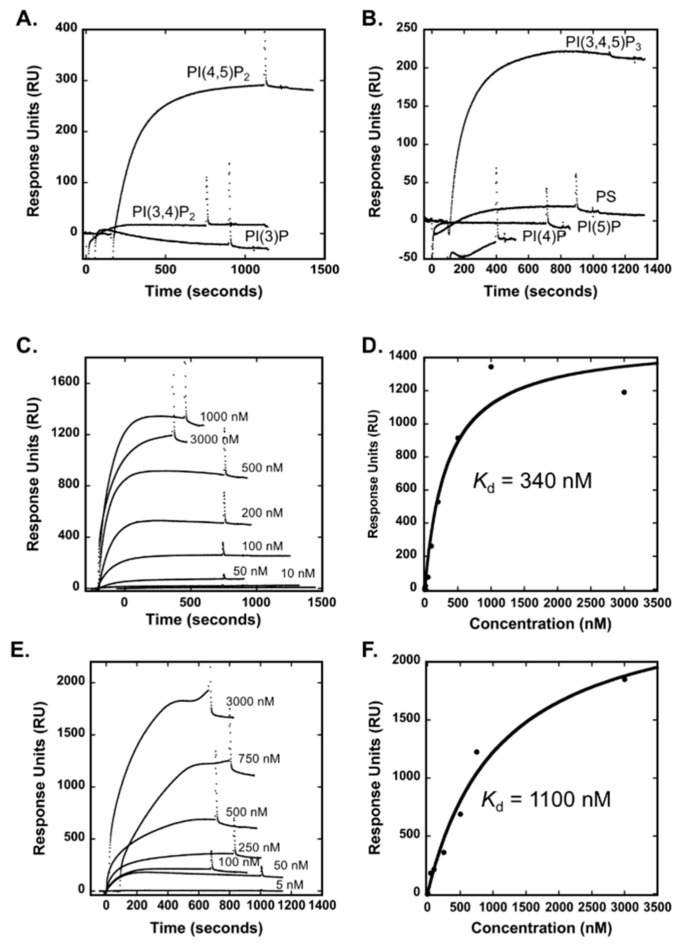
Surface plasmon resonance (SPR) indicates the Smurf1 C2 domain binds to vesicles containing bi-phosphorylated or tri-phosphorylated PIP. (**A**) The Smurf1 C2 domain was injected over a POPC:POPE (80:20) control vesicle surface or an active surface containing (75:20:5, POPC:POPE:PIP (PI(3)P, PI(3,4)P_2_, or PIP_3_) to determine the change in response units (RU) for 100 nM Smurf1 C2 domain. (**B**) The Smurf1 C2 domain was injected over a POPC:POPE (80:20) control vesicle surface or an active surface containing (75:20:5, POPC:POPE:PIP (PI(4)P, PI(5)P, or PIP_3_) or POPC:POPE:POPS (60:20:20). The change in response units (RU) for 100 nM Smurf1 C2 domain was determined by subtracting the sensorgram for binding to the control surface. (**C**) To calculate the apparent affinity of the Smurf1 C2 domain for vesicles containing POPC:POPE:PI(4,5)P_2_, the Smurf1 C2 domain was injected at increasing concentrations (10–3000 nM) and the RU at saturation was determined by subtracting the control sensorgram for each Smurf1 C2 concentration. (**D**) The apparent affinity for vesicles containing PI(4,5)P_2_ was calculated by plotting the saturation resonance unit (RU) value versus Smurf1 C2 domain concentration and fitting with *R*_eq_ = *R*_max_/(1 + *K*_d_/[P]). (**E**) The apparent affinity of the Smurf1 C2 domain for vesicles containing PIP_3_ was determined as in C by injecting increasing concentrations of the C2 domain (5–3000 nM) and determining the saturation response. (**F**) As in D, the saturation response was plotted against the protein concentration to determine the apparent affinity of the Smurf1 C2 domain for vesicles containing PIP_3_. A flow rate of 5 μL/min was used in all experiments.

SPR was next employed to monitor binding of the Smurf1 C2 domain to anionic lipids in LUVs. LUVs, which contain a bilayer serve as mimetics of biological membranes where the LUVs are captured intact on the L1 sensor chip surface. The strongest binding in vitro was to PI(4,5)P_2_ and PIP_3_ (Figure 2A,B) in comparison to PI(3)P, PI(4)P, PI(5)P, PI(3,4)P_2_ or PS. The apparent binding affinity (*K*_d_) of Smurf1 C2 to LUVs containing PI(4,5)P_2_ or PIP_3_ was confirmed by SPR spectroscopy (Figure 2C-F), which demonstrated an apparent affinity of 340 nM for lipid vesicles containing 5 mol% PI(4,5)P_2_ and 1100 nM affinity for vesicles containing PIP_3_. In contrast, Smurf1 C2 displayed weaker binding affinity to other PIPs tested, including PI(3)P, PI(4)P, PI(5)P, and PI(3,4)P_2_. Thus, the LUVs, which are a more representative mimetic of a membrane bilayer allowed for greater selectivity of PI(4,5)P_2_ and PIP_3_ in comparison to the MLV method. Overall, Smurf1 C2 associates with anionic membranes with some selectivity for PI(4,5)P_2_ and PIP_3_ in bilayer membranes. 

### 3.2. Smurf1 C2 Localizes to the Plasma Membrane and Intracellular Anionic Membranes

**Figure 3:**
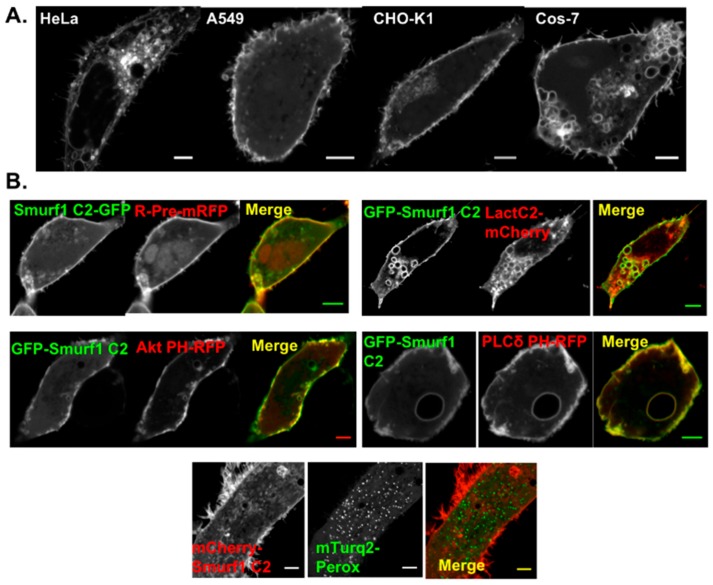
The Smurf1 C2 domain localizes to the plasma membrane and intracellular membranes. Cell lines were transfected with Smurf1 C2 fluorescent fusion constructs to analyze cellular membrane localization. (**A**) GFP-Smurf1 C2 was introduced into HeLa, A549, CHO-K1 and COS-7 cells. Intracellular vesicle localization was observed in all cell types in addition to consistent plasma membrane localization. The Smurf1 C2 domain was also detected in cellular protrusions (*n* ≥ 20 for all cell types). (**B**) In CHOK-1 cells, the GFP-Smurf1 C2 domain co-localizes with R-Pre, a peptide that binds anionic membranes, as well as Lact C2-mCherry, a phosphatidylserine-binding protein at the plasma membrane and intracellular sites, and the PI(4,5)P_2_-binding PLCδ PH-RFP; in HEK293 cells, the GFP-Smurf1 C2 domain co-localizes with the PIP_3_-binding Akt PH-GFP (*n* ≥ 10 for all co-localization experiments). In HeLa cells, mCherry–Smurf1 C2 does not co-localize with the mTurquoise2–peroxisome probe.

In order to determine the localization of the Smurf1 C2 domain in mammalian cells, we generated EGFP and mCherry–Smurf1 constructs for transfection. Four different cell lines were transfected with the mCherry–Smurf1 C2 construct, and in each case the C2 domain localized to the plasma membrane (Figure 3). Localization was also observed in tubular cellular protrusions, probably reflecting interactions with the Smurf1 binding partner, RhoA [10]. Interestingly, we also observed localization to small, intracellular sites. The nature of these membranes was determined by co-transfecting cells with Smurf1 C2 and several lipid-binding probes with well-characterized interactions. Precise co-localization was observed with R-Pre, a peptide marker of anionic membranes, and lactadherin C2, a known PS-binding protein (Figure 3B). These findings indicate that Smurf1 C2 acts in part as a charge sensor by binding to anionic membranes containing PIPs and/or PS [32]. In addition, co-localization with the plasma membrane was observed with the pleckstrin homology (PH) domains of Akt, a PIP_3_-binding protein, and phospholipase Cδ (PLCδ), a well-characterized PI(4,5)P_2_-binding protein (Figure 3B). These findings support the ability of Smurf1 C2 to bind PI(4,5)P_2_- and PIP_3_-containing membranes in vitro. The nature of the intracellular vesicles associated with the Smurf1 C2 domain was investigated further by co-transfecting cells with Smurf1 C2 and organelle markers. We didn’t observe detectable co-localization with mitochondrial or peroxisomal markers (Figure 3B).

### 3.3. Analysis of C2 Mutants Indicates the Loop Region is Necessary for Membrane Localization

**Figure 4:**
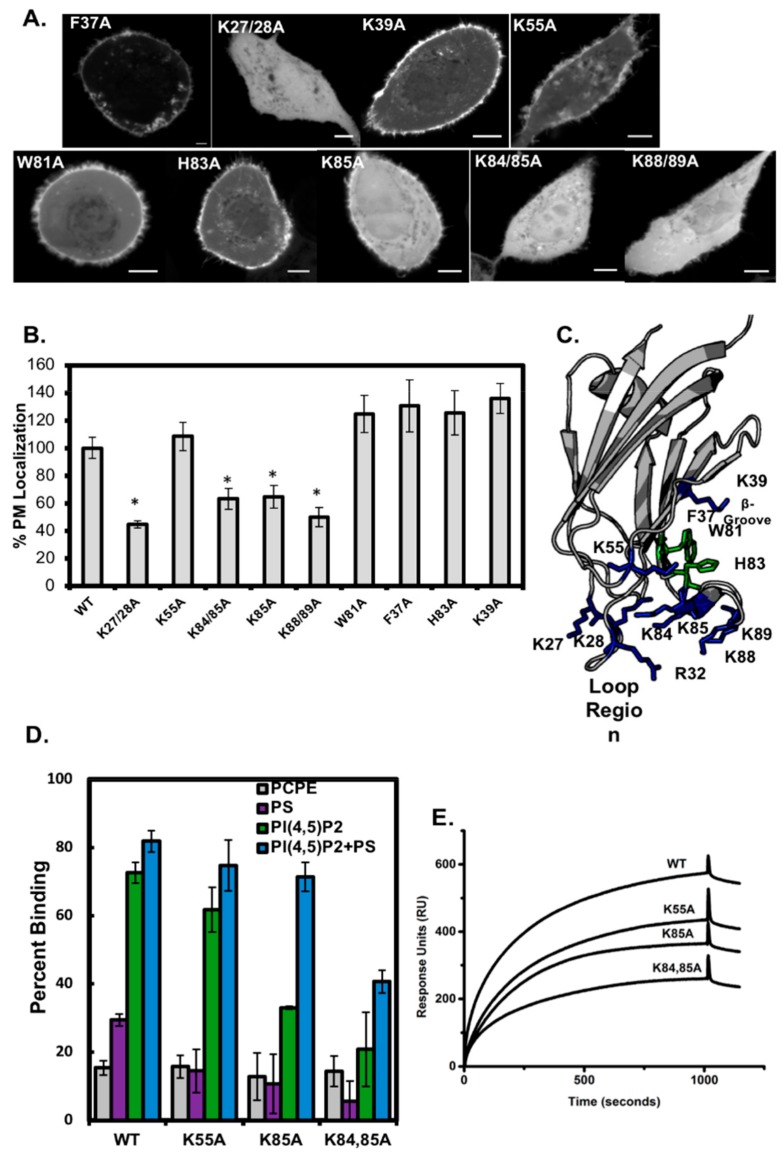
The analysis of Smurf1 C2 lipid-binding mutants demonstrates that the C2 domain loop region is essential for membrane localization and PIP binding. (**A**) The mCherry–Smurf1 C2 domain and the corresponding mutants were introduced into A549 cells to detect changes in plasma membrane localization. (**B**) The %PM localization was determined for all Smurf1 C2 domain mutations shown in A, with the wild-type Smurf1 C2 domain normalized to 100%. A one-way ANOVA was performed (* *p* < 0.008) to determine statistically significant differences. (**C**) Structure of the Smurf1 C2 domain (PDB ID: 3PYC) with the loop region and β-groove mutation residues highlighted. (**D**) A MLV binding assay was performed (Figure 1) for wild-type, K55A, K85A and K84/85A Smurf1 C2 domain using control vesicles (POPC:POPE, 80:20) and those containing PS (POPC:POPE:POPS, 60:20:20), PI(4,5)P_2_ (POPC:POPE:PI(4,5)P_2_, 75:20:5) or PS+PI(4,5)P_2_ (POPC:POPE:POPS:PI(4,5)P_2_ 55:20:20:5). The percent binding (centrifugation assay supernatant and pellet analysis) is plotted as shown. (**E**) Representative SPR sensorgrams are shown for wild-type, K55A, K85A and K84/85A injected over a POPC:POPE:PI(4,5)P_2_ (75:20:5) vesicle surface demonstrating a reduction in binding for all mutations, with K84/85A showing the most significant reduction in line with the vesicle sedimentation and cellular imaging assays.

To determine which amino acid residues mediate the PIP binding and membrane localization of Smurf1 C2, we generated point mutations by site-directed mutagenesis (Figure 4). Following the transfection of mammalian cells with a panel of mutants, we found that the residues targeting Smurf1 C2 to the plasma membrane are located primarily in the loop region of the protein, including Lys27, Lys28, Lys84, Lys85, Lys88 and Lys89. Interestingly, we found that Arg32 and Lys55, which are located towards the back of the loop region on loops β1-β2 and β3-β4, respectively (Figure 4C) are both essential for membrane localization. On the other hand, although mutations within the β-surface cationic patch region (F37A, K39A, and W81A) disrupted membrane localization to a lesser degree, partial to full membrane localization was nevertheless observed. This indicates that the loop region is probably the main membrane-binding surface of Smurf1 C2. This hypothesis was supported by vesicle sedimentation assays and SPR-based MLV-binding experiments with Smurf1 C2 mutants purified for in vitro analysis (Figure 4D,E). The analysis of MLV binding revealed the significantly lower binding activity of mutants K84A and K84/85A to MLVs containing 5 mol% PI(4,5)P_2_ (Figure 4C). In contrast, mutant K55A and wild-type Smurf1 C2 bound to PI(4,5)P_2_ MLVs in a similar manner, agreeing with the ability of K55A to target the plasma membrane in cell-based imaging assays. Notably, association with vesicles containing 20 mol% PS was diminished by all three mutations, but the selectivity for PI(4,5)P_2_ was partially retained in all cases (Figure 4D). Additionally, the combination of PS and PI(4,5)P_2_ resulted in near-wild-type binding activity for K85A but not the double mutant K84/85A, indicating that a strongly anionic membrane interface is sufficient to recruit the Smurf1 C2 domain lacking a cationic residues in the loop region. These binding assays also show that although all the positively charged residues may be required for the general affinity of Smurf1 C2 for anionic PS-enriched membranes, any non-mutated positively charged residues that remain probably contribute to the interactions with PIP. 

### 3.4. Smurf1 C2 Localization to the Plasma Membrane Is PIP Dependent

**Figure 5:**
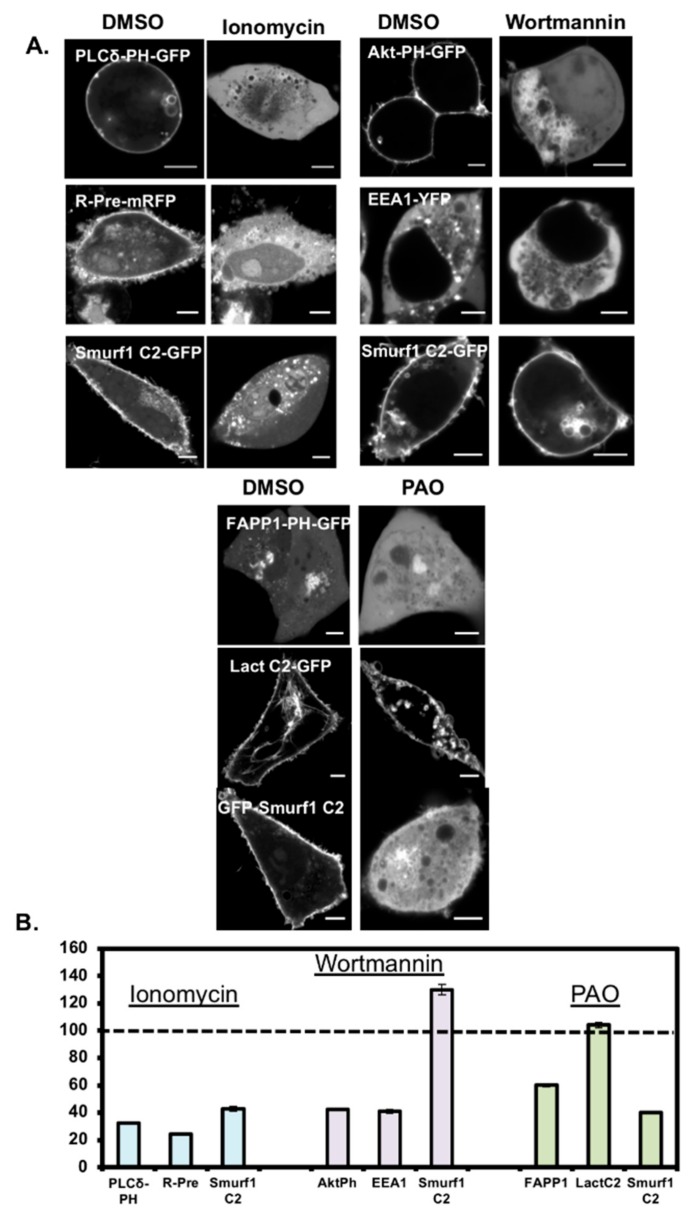
Pharmacological modulation of cellular lipids supports the interaction of Smurf1 C2 with cellular phosphoinositides. (**A**) Ionomycin treatment disrupts the normal localization of Smurf1 C2, PLCδ-PH and R-pre PM, causing the rapid translocation of Smurf1 C2 away from the plasma membrane of CHO-K1 cells (images were taken 2 min after ionomycin treatment). In contrast, wortmannin did not cause any significant dissociation of Smurf1 C2 from the plasma membrane in HEK293 cells. Exposure to phenylarsene oxide for 20 min also caused the dissociation of Smurf1 C2 from the plasma membrane, probably due to the loss of PI(4)P, a direct precursor of PI(4,5)P_2_. The Golgi localization of the PI(4)P-binding FAPP1 PH domain was dramatically reduced by treatment with phenylarsene oxide, whereas there was no effect on the PS-binding lactadherin C2. (**B**) Plasma membrane localization was quantified for the Smurf1 C2 domain and lipid reporter constructs for ionomycin, wortmannin, and PAO treatments (*n* ≥ 4 for all experiments). Scale bars = 5 μm.

We established the principal determinant of Smurf1 C2 interactions with cell membranes by applying pharmacological reagents to mammalian cells causing the depletion of PIPs and PS (Figure 5). Ionomycin induces phospholipase C activity, thus causing PI(4,5)P_2_ metabolism and the loss of PS asymmetry across the plasma membrane by the inhibition of flippases and activation of scramblases. Following treatment, we found that the PH domain of PLCδ diffused away from the plasma membrane and re-localized in the cytosol. The same phenomenon was observed for R-Pre, a peptide that binds to anionic membranes. Ionomycin also caused the Smurf1 C2 domain to diffuse away from the plasma membrane, but we observed no negative effect on intracellular staining (indeed, intracellular binding may even have increased following treatment). This indicates that, although the lipid profile of the plasma membrane was disrupted, the remaining intracellular PIP and anionic lipid targets were sufficient to retain Smurf1 C2 binding. These findings confirmed that either PIPs or PS in the plasma membrane of mammalian cells is sufficient for interactions with Smurf1 C2.

The role of PIPs in Smurf1 C2 membrane localization were identified by treating cells with wortmannin, a specific inhibitor of phosphoinositide 3-kinase, to reduce the levels of PI(3)P, PI(3,4)P_2_ and PIP_3_. In treated HEK293 cells, the PIP_3_-coordinating Akt PH domain diffused away from the plasma membrane and the PI(3)P-coordinating EEA1 FYVE domain diffused away from the endosomes. Smurf1 C2 remained bound to the plasma membrane, indicating that the major determinant of plasma membrane association is not PIP_3_. Accordingly, we then applied the reducing agent phenylarsene oxide for 30 min at 37 °C to inhibit phosphoinositide 4-kinases. Following this treatment, the PI(4)P-coordinating protein FAPP1 and the PI(4,5)P_2_-coordinating PH domain of PLCδ (not shown) dissociated from the Golgi body and plasma membrane, respectively. Lactadherin C2, a PS-binding C2 domain, remained bound to the membrane. Like FAPP1 and the PH domain of PLCδ, Smurf1 C2 dissociated from the plasma membrane and other cellular membranes. These data support the hypothesis that PI(4,5)P_2_ is the main determinant responsible for the plasma membrane localization of Smurf1 C2, although the contribution of PI(4)P cannot be excluded.

**Figure 6:**
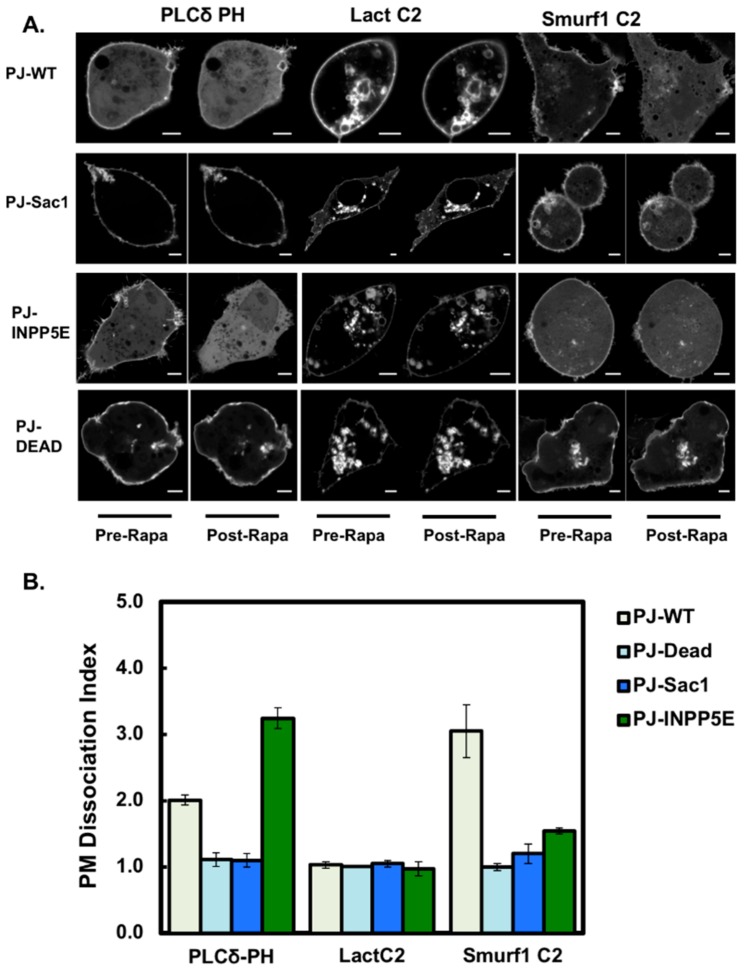
Rapamycin-recruited phosphatases knock down plasma membrane phosphoinositides to induce the de-localization of Smurf1 C2. (**A**) Images of controls (PLCδ-PH-GFP and Lact C2-mCherry) in addition to Smurf1 C2-GFP were taken before and 5 min after the addition of rapamycin. (**B**) Quantification of PIP depletion presented as dissociation indices (ratio of *I_PM_*/*I_cyto_* before and after treatment) for PLCδ-PH, lactadherin C2, and Smurf1 C2 (data are means ± SEM, *n* ≥ 4 different cells). The wild-type pseudojanin construct (PJ-WT) is compared to PJ-DEAD, PJ-Sac1, and PJ-INPP5E activity. These results show that Smurf1 is displaced following PIP depletion, showing that it binds to PIP at the plasma membrane. Scale bars = 5 μm.

More information concerning the specificity of PIP binding was gained from real-time PIP depletion studies [24]. Using the dual 4-phosphatase/5-phosphatase wild-type pseudojanin (PJ-WT) and its catalytically inactive counterpart PJ-DEAD, we found that the Smurf1 C2 domain de-localized from the plasma membrane following the rapamycin-induced depletion of PIPs via the recruitment of PJ-WT to the plasma membrane (Figure 6). Dissociation was measured as a dissociation index (see methods section). Whereas the de-localization of Smurf1 C2 from the plasma membrane was definitive, as was the case for the PI(4,5)P_2_-selective PH domain of PLCδ, lactadherin C2 remained fully localized to the plasma membrane, indicating that PIP binding is the primary driver of interactions between the plasma membrane and Smurf1 C2. 

We then investigated whether PI(4)P and/or PI(4,5)P_2_ are responsible for the localization of Smurf1 on the plasma membrane by using (i) the catalytically inactive 4-phosphatase construct with an active 5-phosphatase (PJ-INPP5E), which depletes PI(4,5)P_2_ and PIP_3_ but leaves PI(4)P intact; and (ii) the PJ Sac1 construct with a catalytically inactive INPP5E, which depletes PI(4)P but leaves PI(4,5)P_2_ intact. The former caused the PH domain of PLCδ to diffuse away from the plasma membrane whereas the latter maintained the interaction. We found that Smurf1 C2 dissociated from the plasma membrane following the recruitment of PJ-WT but was retained at the membrane following the recruitment of PJ-Sac1, indicating that PI(4,5)P_2_ can interact with the Smurf1 C2 domain. Remarkably, when PI(4,5)P_2_ was depleted, Smurf1 C2 was partially retained at the membrane, whereas the PH domain of PLCδ dissociated almost completely (Figure 6A,B). Therefore, Smurf1 C2 appears to be retained at the plasma membrane by the residual PI(4)P and PS. This agrees with our in vitro data showing that Smurf1 C2 has a broad selectivity for PIPs and can coordinate more than one PIP head group in the cellular setting. The PS in the plasma membrane is also likely to play a significant role when the levels of PI(4,5)P_2_ or PI(4)P are selectively depleted. 

### 3.5. RFP-Ubiquitin Localization is Dependent on the Ligase Activity of Smurf1 at the Membrane

**Figure 7:**
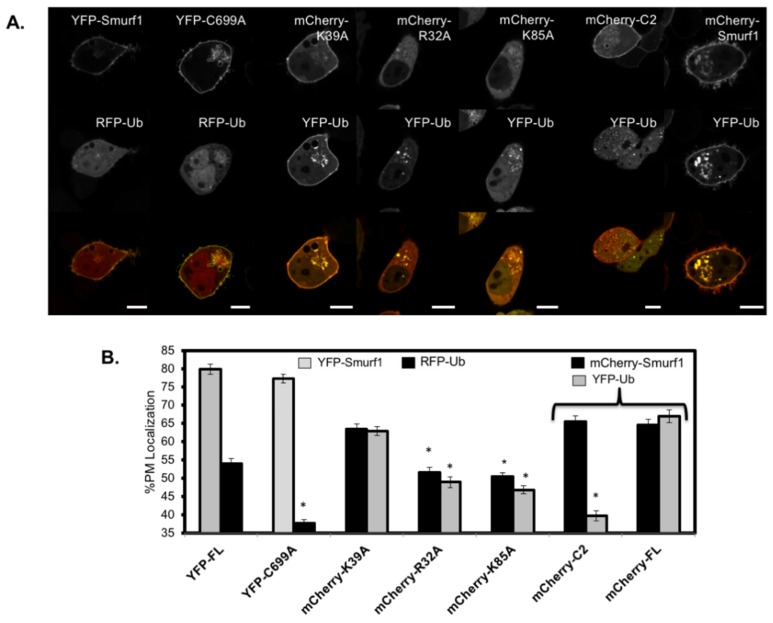
C2 domain loop residues regulate Smurf1 plasma membrane ubiquitylation. (**A**) Representative images of Smurf1 (wild-type and mutants) and ubiquitin. HEK293 cells were co-transfected with the indicated Smurf1 and ubiquitin constructs and imaged on a confocal microscope 24 h post-transfection. The %PM localization of Smurf1 and ubiquitin constructs was quantified using ImageJ software (see methods). Between 9-17 cells were analyzed per co-expression experiment from three independent transfections. (**B**) Percent plasma membrane localization of Smurf1 (wild-type and mutants) and ubiquitin. A Two-way ANOVA was performed to evaluate statistically significant changes in ubiquitin or Smurf1 %PM localization with respect to WT co-transfection values (YFP-Smurf1/RFP-Ub or mCherry–Smurf1/YFP-Ub). * *p* < 0.001. Error bars represent SEM. Scale bars = 10 μm.

To assess the functional consequence of C2 domain mutations in full-length Smurf1, a live cell ubiquitination assay was designed using cells co-transfected with constructs encoding full-length Smurf1 and N-terminally tagged ubiquitin, the latter carrying a fluorescent tag but maintaining its capacity to be conjugated to proteins and recycled like endogenous ubiquitin [1,2]. When cells were co-transfected with wild-type YFP-Smurf1 and RFP-ubiquitin, the fluorescent signals representing both proteins were enriched in the plasma membrane (Figure 7A). To ensure that the localization of ubiquitin was dependent on Smurf1 ubiquitin ligase activity and not an alternative means of recruitment, cells were co-transfected with the catalytically inactive YFP-Smurf1-C699A construct and RFP-ubiquitin. In these cells, YFP-C699A was still localized to the plasma membrane, like its wild-type counterpart, but RFP-ubiquitin was no longer recruited (Figure 7A,B). Instead, RFP-ubiquitin was re-localized to the cytoplasm, with strong enrichment in the nucleus, similar to the typical localization of ubiquitin. This indicates that the localization of RFP-ubiquitin is dependent on the ligase activity of Smurf1 at the membrane. This assay also revealed a characteristic reorganization of ubiquitin in the cell due to Smurf1 activity, providing a spatial profile for Smurf1 ubiquitination with strong activity at the plasma membrane. An alternative approach using mCherry–Smurf1 and YFP-ubiquitin yielded similar results, with YFP-ubiquitin co-localized at the plasma membrane (Figure 7A,B).

We then repeated the assay with several Smurf1 mutants in the mCherry vector to assess the role of the C2 domain in Smurf1 recruitment to the plasma membrane and substrate ubiquitination. The localization and substrate ubiquitination activity of the C2 domain mutation mCherry-K39 was comparable to wild-type Smurf1, confirming the β-groove is not responsible for targeting Smurf1 to the plasma membrane (Figure 7A,B). In contrast, two mutants with substitutions in the loop region of the C2 domain (K85A and R32A) showed a loss of plasma membrane localization and membrane-associated ubiquitination. It is important to note that both K85A and R32A still showed catalytic activity, as indicated by the reduced enrichment of ubiquitin in the nucleus, a marker of free ubiquitin depletion [2]. As expected, when the mCherry–Smurf1 C2 domain alone was expressed with YFP-ubiquitin, no ubiquitination was detected at the plasma membrane and YFP-ubiquitin showed strong nuclear localization. This demonstrates that the C2 domain regulates the localization of Smurf1 at the plasma membrane and membrane-associated ubiquitination, and that loop residues implicated in PIP coordination play a key role in the ubiquitination of targets at the plasma membrane. 

**Figure 8:**
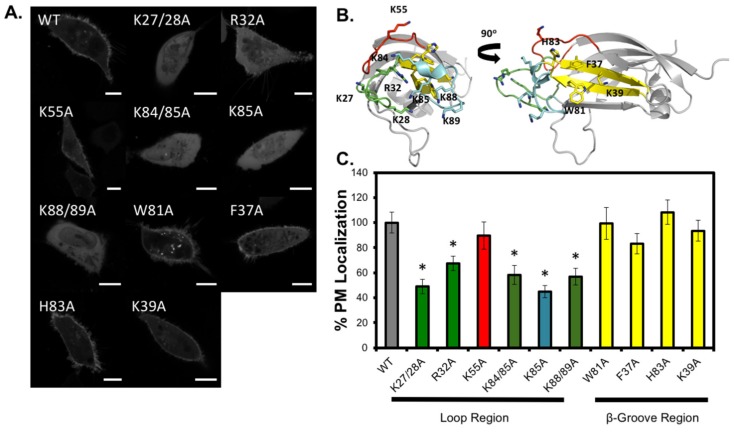
The C2 domain loop region, not the β-groove, is essential for Smurf1 plasma membrane localization. (**A**) Representative images of mCherry–Smurf1 fusion constructs (wild-type and mutants in the C2 domain of the full-length construct). A549 cells were transfected with the indicated mutant Smurf1 full length constructs and imaged on a confocal microscope 24 h post-transfection. Between 17-23 cells were analyzed per mutant from three independent transfections. (**B**) The Smurf1 C2 domain crystal structures is depicted in cartoon form (PDB ID: 3PYC) to highlight mutated residues in the loop regions or the β-groove. (**C**) %PM localization was quantified with a MATLAB script. %PM localization = Ave PM fluorescence/(Ave PM fluorescence + Ave cytoplasm fluorescence) × 100%. Calculated values are normalized to WT. A one-way ANOVA was utilized to ascertain statistically significant changes in %PM localization with respect to WT. * *p* < 0.01. Error bars represent SEM. Scale bars = 10 μm.

To gain further insight into the C2 domain residues required for the localization of full-length Smurf1 to the plasma membrane, we evaluated the localization of mCherry fusion constructs based on wild-type Smurf1 and mutants in the C2 domain loop regions or β-groove (Figure 8A,B). In agreement with the isolated Smurf1 C2 domain studies, Smurf1 proteins with single or double mutations in the cationic loops (K27/28A, R32A, K84/85A, K85A or K89A) showed a significant reduction in localization to the plasma membrane. In contrast, those with mutations in the β-groove (F37A, K39A, W81A and H83A) showed no significant dissociation from the plasma membrane, and similar results were observed for mutations affecting K55, a cationic residue located just behind the cationic loop region. These findings confirm that residues in the cationic loop region (K27, K28, R32, K84, K85, K88 and K89) are the main drivers of plasma membrane localization via binding to PIP. 

### 3.6. Computational Analysis of PIP Binding Reveals Two Potential Binding Orientations

**Figure 9:**
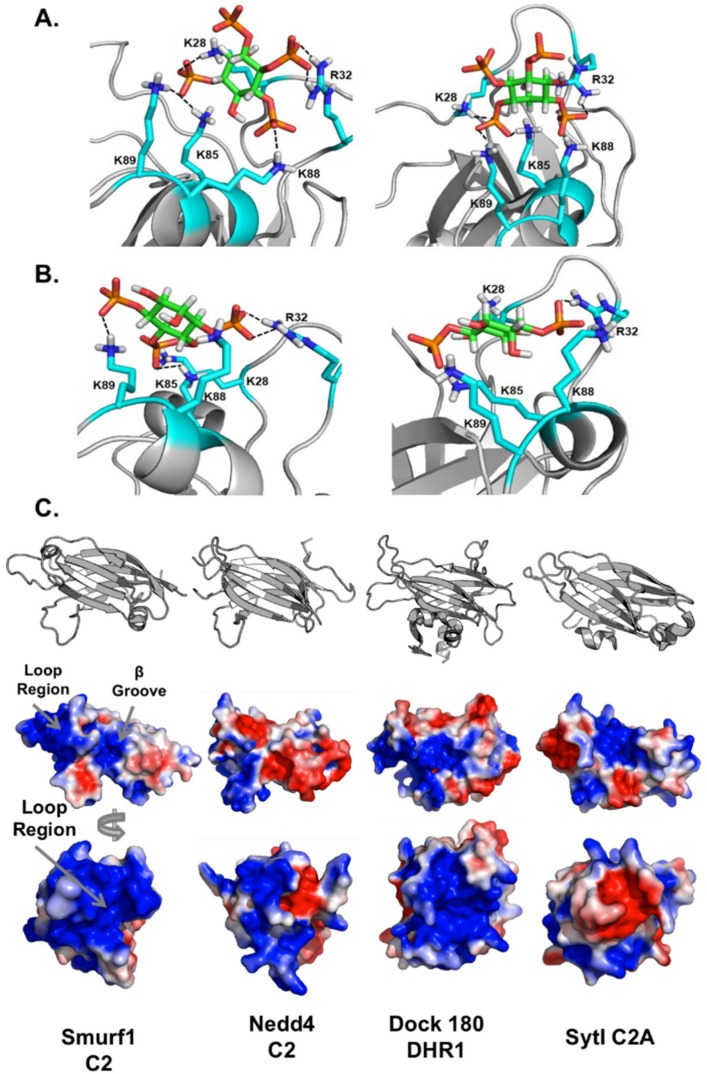
Molecular dynamics and inositol phosphate docking reveals potential PIP coordination mechanisms for the Smurf1 C2 domain. (**A**) The Smurf1 C2 domain is shown docked with inositol(1,3,4,5)P_4_ indicating potential interactions between R32 and the 4-phosphate, K28, K85 and K89 with the 1-phosphate, and K88 with the 5-phosphate (left panel). The right panel shows a second possible orientation, with R32 and K88 interacting with the 1-phosphate, K28, K85 and K89 interacting with the 3-phosphate, and the 4-phosphate and 5-phosphate exposed to the solvent. (**B**) In the left panel, the Smurf1 C2 domain is shown with a potential binding orientation of inositol(1,4,5)P_3_. Here, R32 and K88 interact with 1-phosphate whereas K89 interacts with 4-phosphate, and K28 and K85 interact with 5-phospate. The right panel shows a docking orientation of the Smurf1 C2 domain and inositol(1,4)P_2_, where R32 and K88 interact with 1-phosphate, K85 and K89 interact with 4-phosphate, and K28 interacts with the 6-hydroxyl group (**C**) The top panel shows the overall structure of Smurf1 C2 (charge ~ +9), Nedd4-1 C2 (charge ~ +1), DOCK180 DHR1 C2-like (charge ~ −1), and synaptotagmin I C2A (charge ~ +2) to compare potential loop and β-groove PIP binding mechanisms (PDB IDs: Smurf1 C2 = 3PYC, Nedd4 C2 = 2NSQ, Dock180 C2 = 3L4C, Syt1C2A = 3GPE). The electrostatic solvent-accessible surface of the C2 domains was generated using the Mac Pymol APBS plugin. The side of the C2 domain with the canonical cationic patch is represented in cartoon form and with the electrostatic surface (middle panel) and the C2 domains were then rotated 90° to the right on the horizontal axis to observe the protein from the loop region (bottom panel).

To understand how PIPs bind to Smurf1 C2, we compared computational models, generated using a sequence of docking and molecular dynamics calculations, with our experimental results. The simulations indicated two possible binding orientations for inositol(1,3,4,5)P_4_ that agreed with the experimental data. In the first orientation (Figure 9A, left), R32 interacts with the 4-phosphate, K28, K85 and K89 interact with the 1-phosphate, K88 interacts with the 5-phosphate, and the 3-phosphate is exposed to the solvent rather than interacting with the C2 domain. In the second orientation (Figure 9A, right), R32 and K88 interact with the 1-phosphate, K28, K85 and K89 interact with the 3-phosphate, and the 4-phosphate and 5-phosphate do not interact with the protein and are exposed to the solvent. Both orientations explain how the R32A, K85A and K88/89A mutations interfere with binding. Regarding the double mutant K27/28A, our simulation shows that only K28 interacts with inositol(1,3,4,5)P_4_, whereas K29 and K84 do not interact with the ligand. Hence, the effect of the K84/K85A double mutation is mainly caused by K85A. Importantly, the simulation did not indicate which of the two proposed binding orientations was more feasible. We also investigated the molecular dynamics of Smurf1 binding to inositol(1,4,5)P_3_ (Figure 9B, left) and inositol(1,4)P_2_ (Figure 9B, right). Again, we found that K28, R32, K85, K88 and K89 interact with both ligands. Specifically, for inositol(1,4,5)P_3_, R32 and K88 interact with 1-phosphate whereas K89 interacts with 4-phosphate, and K28 and K85 interact with 5-phospate. For inositol(1,4)P_2_, R32 and K88 interact with 1-phosphate, whereas K85 and K89 interact with 4-phosphate, and K28 interacts with the 6-hydroxyl group. 

## 4. Discussion

In this study, we investigated the membrane localization of the Smurf1 C2 domain at the molecular level. We found that membrane localization is driven primarily by PI(4,5)P_2_, which binds to the loop region of the C2 domain—not to the canonical cationic β-groove, as is the case for many other C2 domains, including PKCα, synaptotagmins and rabphilins [22,33,34,35,36]. We found that Smurf1 C2 binds anionic lipids with selectivity for PIPs, can target multiple cellular sites, and acts as a sensor of anionic membranes. We demonstrated the affinity of the Smurf1 C2 domain for several PIP species using MLVs and a sedimentation assays and LUVs using SPR spectroscopy. The addition of PS increased binding affinity by up to 50% in the MLV assays even though the C2 domain showed minimal binding to PS alone. These results suggest that Smurf1 can detect the coincidence, or at least increased anionic charge density, of PIPs and PS in membranes. Several C2 domains have previously been shown to coordinate both PS and PI(4,5)P_2_, and such a mechanism for C2 domains is therefore well documented [36,37]. 

Careful examination of the Smurf1 C2 domain structure is required to understand the basis of lipid binding in vitro and in cells. Interestingly, the loop region of the protein appears to be the main driver of membrane localization. The Smurf1 C2 loop region is more positively charged than the corresponding region of other C2 domains, except a small region in which a non-polar surface is shaped by Phe30 and Phe31, hydrophobic residues that are likely to penetrate the membrane bilayer (Figure 9). Although it is clear that calcium-binding residues in the loop regions of the synaptotagmin and PKCα C2 domains are not present in Smurf1, neither are the positively charged β-groove residues. The synaptotagmin C2B domain binds PI(4,5)P_2_ selectively via its cationic patch region, whereas the C2A domain does not [22]. The main difference between the C2A and C2B domains in this region is the presence of a glutamic acid residue in C2B replacing a key lysine residue in C2A, a change that abolishes the interaction with PI(4,5)P_2_ [22]. Reversal of this charge in the E194K mutant was shown to re-establish PI(4,5)P_2_ coordination and increase inositol head group affinity by almost 20-fold. This finding highlights the need to conserve three or four positively charged residues in the β-groove in order to coordinate PIP head groups. Smurf1 C2 has only one positive residue in this region (Lys39), emphasizing the low probability that PIP binding involves the β-groove. These results are in agreement with our analysis of C2 mutants, in which the mutation of Phe37 and Lys39 in the β-groove region did not significantly interfere with membrane interactions.

Few C2 domains have been shown to bind membranes via the loop region to coordinate multiple PIP species. We found that Smurf1 C2 has novel PIP-binding properties in that it coordinates PIPs mainly via loop region residues. In Smurf1, the surface has a strong electrostatically positive potential (Figure 9), whereas the corresponding regions of PKCα, Nedd4-1 C2, and synaptotagmin I C2B have near-neutral loop region surfaces (–2 and +1) in the absence of calcium. The C2-like Dock 180 protein and Smurf1 C2 surfaces both have loop regions with an overall charge of +6. This is relevant in the light of recent studies showing that Dock180 coordinates PIPs in its loop region. Dock 180 is a guanine nucleotide exchange factor for Rac1 and was originally shown to contain a PIP_3_-binding domain termed a dock homology region (DHR) [38]. A C2 core β-sandwich is present, but the DHR-1 domain contains additional loops that distinguish it from canonical C2 domains (Figure 9) and these loops facilitate the coordination of PIP_3_ [39]. The authors speculated that the combination of structural changes in the protein reduces the number of residues specifically coordinating either PI(4,5)P_2_ or PIP_3_, and the higher electropositive nature of the region and surrounding area helps to reduce the specificity of PIP binding. We believe that the Smurf1 C2 domain uses a similar mechanism. In the MLV assays, it is apparent that Smurf1 C2 associates with all PIP species with PIP_3_ and the bis-phosphorylated PIPs exhibiting the greatest association. In the LUV assays using SPR, where LUV bilayers are a more representative mimetic of biological membranes, the Smurf1 C2 domain bound PI(4,5)P_2_ and PIP_3_ with highest affinity. Thus, while the overarching results among the two lipid binding assays are similar, the SPR assays are more in line with cellular data demonstrating Smurf1 and its respective C2 domain dissociated from the plasma membrane more readily when PI(4,5)P_2_ was depleted in comparison to PI(4)P. The overall electropositive nature of the Smurf1 loop region also likely enables it to coordinate multiple PIP species. The large surface area of this region would certainly enable it to coordinate more than one lipid head group, and the two phenylalanine residues present at the outermost tip of the β3-β4 loop could participate in membrane penetration.

We also demonstrated that the lipid-targeting ability of Smurf1 C2 contributes to its localization in several organelles and vesicles. Smurf1 C2 localizes intracellularly in a similar manner to the consensus PS sensor lactadherin C2 and the anionic charge sensor R-Pre and does so in a PIP-dependent manner. The moderate selectivity for individual PIP head groups raises the interesting possibility that Smurf1 C2 can target different cellular membranes based on the relative content of PIP and PS, and the presence of interacting substrates, as is the case for coincidence detection binding proteins such as FAPP1, which binds PI(4)P and Arf1 in the Golgi body [40]. Further studies are required to determine whether Smurf1 targets substrates at intracellular sites based on the localization driven by its C2 domain, and under which favorable conditions such interactions may occur. Interestingly, PS is enriched in regions of the plasma membrane responsible for cell polarization [41]. Given the role of Smurf1 in the maintenance of cell polarity and adhesion, it is tempting to postulate that such PS-enriched regions may prime the membrane for the recruitment of Smurf1 to those sites, especially those sites containing PIPs such as PI(4,5)P_2_ or PIP_3_.

Smurf1 is an E3 ubiquitin ligase that targets several cellular sites and thus diverse substrates, probably conferring the flexibility to bind different membranes in response to substrate availability. Whereas some Smurf1–substrate interactions are mediated by WW domains, others are mediated by the C2 domain itself [42,43,44]. Furthermore, the Smurf1 C2 domain is necessary to target mitochondria suffering oxidative damage to the lysosomal membrane for degradation [16]. The interaction between Smurf1 and mitochondria involves WW domains rather than the C2 domain, and we therefore speculate that anionic lipid selectivity of the C2 domain may help to facilitate the fusion of mitochondrial and lysosomal membranes. The lysosomal membrane is known to contain PI(3)P, which plays a role in the recruitment of other autophagy factors [45] and damaged mitochondria can expose the anionic lipid cardiolipin. Given that the clearance of damaged mitochondria is a characteristic of several neurological pathologies, including Parkinson’s disease, further studies are required to investigate the role of the Smurf1 C2 domain in the fusion of lipid membranes and the ability to target lysosomal membranes. Furthermore, PS and PI(3)P are enriched at sites of phagosome formation and contribute to the targeting of cargo to maturing phagosomes during autophagy [45,46]. Further investigation is therefore required to understand the role of the Smurf1 C2 domain in the targeting of substrates to different intracellular sites, during both physiological [46] and pathophysiological processes [47].

## 5. Conclusions

We have investigated the lipid-binding mechanism of the Smurf1 C2 domain, revealing a general affinity for anionic membranes in addition to selective binding for PI(4,5)P_2_ and PIP_3_ as determined with LUVs and SPR. The Smurf1 C2 domain localized to the plasma membrane as expected, but also to other membranes depending on the PIP content. The C2 domain of this Nedd4 family E3 ubiquitin ligase therefore acts as an anionic charge sensor and a selective PIP-binding domain. We found that the interaction between the Smurf1 C2 domain and membranes/PIPs is primarily directed by the loop region rather than the β-groove and is dependent on the presence of PIPs. The quantity of ubiquitin found at the plasma membrane interface depends on the lipid-binding properties of Smurf1. We have therefore elucidated the mechanism by which Smurf1 targets substrates at cell membranes and we have provided a detailed molecular explanation for the selective binding of the Smurf1 C2 domain to the plasma membrane, which is enriched with anionic lipids including PI(4,5)P_2_ and PS.
